# Supplementary figures and images for: Transcript Variants of Genes Involved in Neurodegeneration Are Differentially Regulated by the APOE and MAPT Haplotypes
Source: Genes (Basel). 2021 Mar 15;12(3):423. doi: 10.3390/genes12030423 (PMC7999745; doi:10.3390/genes12030423)

Figure 1S: Detailed circos plots of all analysed loci

APOE

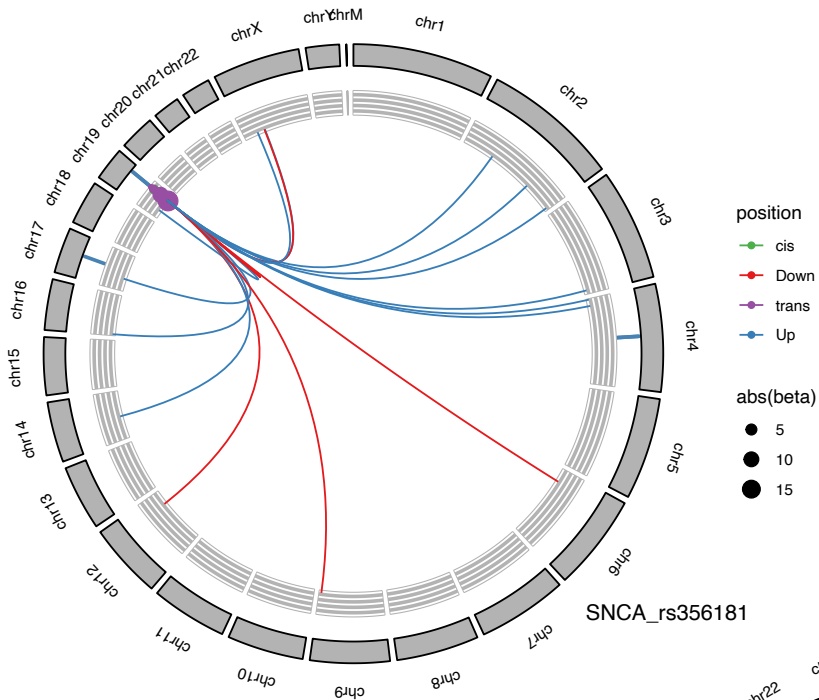

APOE\_e4

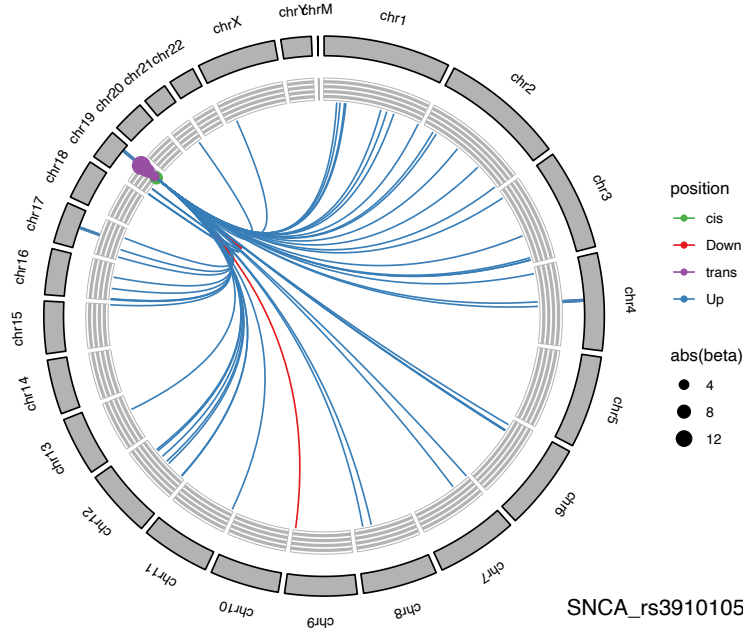

MAPT

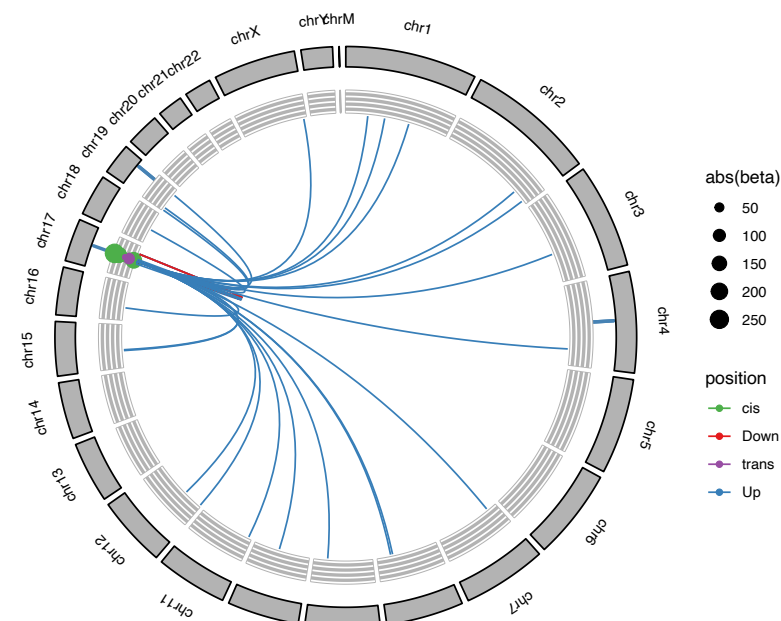

SNCA\_rs356181

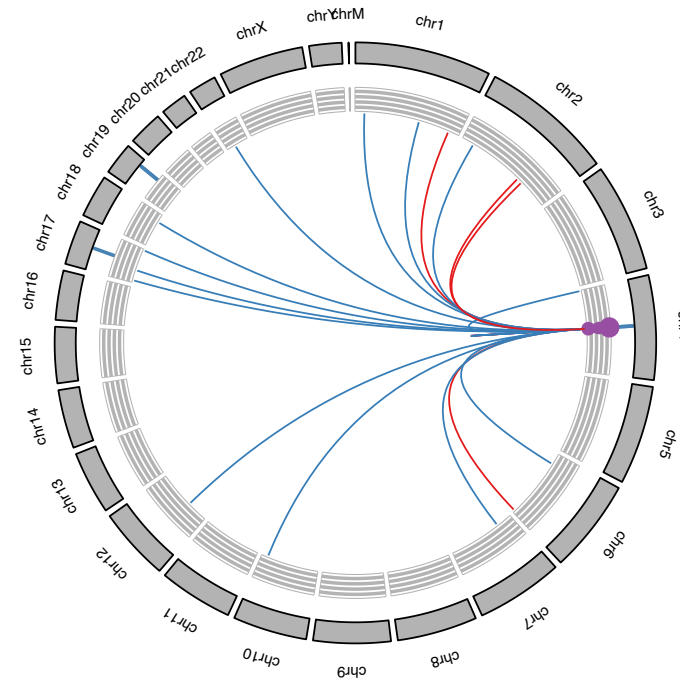

SNCA\_rs3910105

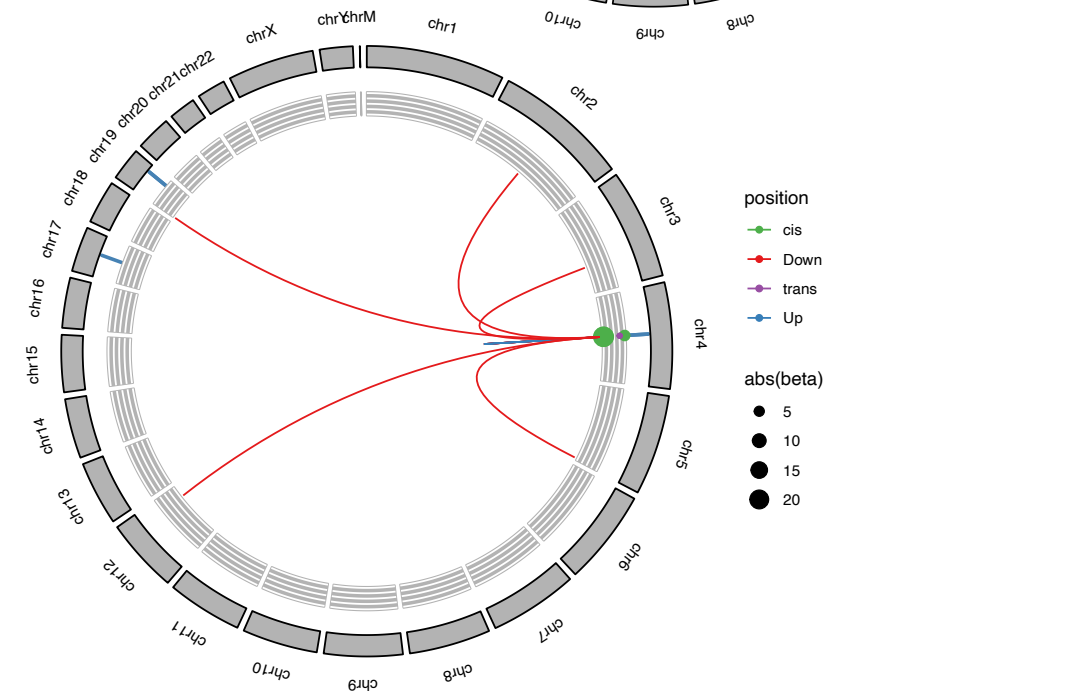

Supplement: Supplementary file 1 [file genes-12-00423-s001.zip › Figure1S_CombinedCircosPlots_SupplementKoksREV.pdf]
